# Effects of the Incorporation of Calcium Chloride on the Physical and Oxidative Stability of Filled Hydrogel Particles

**DOI:** 10.3390/foods11030278

**Published:** 2022-01-20

**Authors:** Xin Li, Chuanai Cao, Dongxue Yuan, Qian Liu, Jinhai Zhao

**Affiliations:** 1 College of Food Science, Northeast Agricultural University, Harbin 150030, China; lixin810910@hotmail.com (X.L.); cca930504@hotmail.com (C.C.); S201001007@neau.edu.cn (D.Y.); 2 Heilongjiang Green Food Science & Research Institute, Harbin 150028, China; 3 Institute of Advanced Technology, Heilongjiang Academy of Science, Harbin 150001, China

**Keywords:** filled hydrogel, CaCl_2_, physical stability, viscoelastic behavior, oxidative stability

## Abstract

In this study, the effects of calcium chloride (CaCl_2_) addition on the physical and oxidative stabilities of filled hydrogel were investigated. The results revealed that CaCl_2_ significantly enhanced the particle size, interfacial layer thickness, apparent viscosity, and viscoelastic behavior of filled hydrogels and decreased their light and whiteness values (*p* < 0.05). This phenomenon was mainly attributed to the strong binding ability between Ca^2+^ and protein/pectin mixtures, which were present in the interfacial area or aqueous phase, as verified by cryo-scanning electron microscopy results. Moreover, lower levels of CaCl_2_ (2 or 4 mM) significantly enhanced the oxidative stability of filled hydrogels (*p* < 0.05), particularly at a concentration of 4 mM. However, a higher level of CaCl_2_ (6 or 8 mM) resulted in an electrostatic shielding effect, which resulted in the aggregation of multiple droplets and the flocculation of the filled hydrogels, which negatively affected the oxidative stability of filled hydrogels. The findings of this study indicated that appropriate Ca^2+^ levels (4 mM) improved the physical and oxidative stability of filled hydrogel, and this finding may provide useful insights for the development of effective delivery systems for specific applications.

## 1. Introduction

Traditionally, emulsion systems are classified as oil-in-water (O/W), water-in-oil-in-water (W/O/W), oil-in-water-in-water (O/W/W), and oil-in-water-in-oil (O/W/O) emulsions. Emulsion systems have attracted widespread attention as a delivery system for the encapsulation or delivery of bioactive compounds into aqueous foods [1,2]. Among these systems, filled hydrogels are considered a typical representative O/W/W emulsion system, and they exhibit considerable application prospects for the delivery of bioactive compounds [3,4]. Filled hydrogels are prepared by directly incorporating an O/W emulsion into a phase-separated-based water-in-water (W/W) emulsion based on two biopolymers (mainly protein and polysaccharide) [5]. Compared to other emulsion systems, filled hydrogels possess unique advantages, such as higher oxidative stability, more effective control of the release of bioactive compounds, and enhancement of the desirable flavor of low-fat foods [2,6,7,8]. For the application of filled hydrogels for the manufacture of food products, it is essential that they can adapt to numerous environmental conditions (e.g., temperature, mechanical mixing speed, pH, and ionic strength) [9]. Matalanis, Lesmes, Decker, and McClements reported that adjusting the pH to 5.0 promoted a shift in their adsorption ability of polysaccharides from continuous phase onto proteins to the dispersed phase around protein-coated droplets [4]. In addition, Cai, Du, Zhu, and Cao reported that sodium chloride addition shields a part of the charge of biopolymers and affected electrostatic interactions between biopolymers, which deteriorated the properties of the final product with respect to lower emulsion stability [10]. Furthermore, owing to the thermodynamic instability of filled hydrogels during a longer storage period, they are susceptible to an oxidative instability phenomenon.

In order to simultaneously improve the physical and oxidative stability of filled hydrogels, the gelling of dispersed or continuous phases of filled hydrogels with various methods, such as adjusting temperature (e.g., cold-induced or heat-induced gelation), adding ions (e.g., calcium ion or potassium ion), or cross-linking agents (such as genipin, transglutaminase, or laccase), has been investigated. Owing to the beneficial biological functions of Ca^2+^ for optimal health and disease prevention, calcium chloride (CaCl_2_) is commonly applied to the food industry [11]. The addition of Ca^2+^ to filled hydrogels results in crosslinking with proteins, change in the protein conformation, enhanced viscosity, and promotion of the gel’s network structure [12,13]. Xiao et al. also reported that appropriate Ca^2+^ levels could induce the full unfolding and orderly aggregation of protein molecules, thus changing the interactions between protein and polysaccharide in mixtures [14]. Moreover, the addition of Ca^2+^ has a significant influence on the stability of the emulsions. Du, Ji, Lyu, Liu, and Ding reported that the addition of Ca^2+^ into emulsions could alter the surface charge density of droplets and the adsorption properties of the interface film, thereby improving the stability of the emulsion [14]. Ren et al. also showed that the incorporation of Ca^2+^ could increase the continuous phase viscosity and alter the equilibrium between ordered and disordered states of citrus pectin chains, thus resulting in the formation of large emulsion droplets in the emulsions of citrus oil emulsions by citrus pectin [15]. In our previous study, filled hydrogels were successfully fabricated by mixing protein-stabilized O/W emulsion with the phase separation product based on heat-denatured whey protein concentrates (HWPCs) and high methoxy pectin (HMP) [3]. HMP is an anionic polysaccharide and has been widely applied in the food industry due to its gelling and thickening properties. Moreover, HWPC was selected owing to its ability to expose more hydrophobic and sulfhydryl groups buried in the whey protein when whey protein is subjected to heating treatment over denaturation temperatures, which could be ascribed to phase separation behavior in protein–polysaccharide systems containing [16]. Results showed that when the mass ratio of HWPC and HMP was 3:1, the filled hydrogels had the best physical properties and oxidative stability. However, studies on the effect of Ca^2+^ addition on the stability of filled hydrogels are rare. Thus, the objective of the current study was to determine the effect of CaCl_2_ levels on the physical properties and lipid and protein oxidative stabilities of filled hydrogels. The results of this study may provide useful insights on the adjustment of the functional properties of specific functional foods.

## 2. Materials and Methods

### 2.1. Materials

Whey protein concentrate (WPC) was purchased from Hilmar Ingredients Co. Ltd. (Hilmar, CA, USA), and the protein content was 80% (*w*/*w*) based on total weight. High methoxy pectin (HMP) was purchased from Yuxincheng Biotechnology Co. Ltd. (Kaifeng, China), and the esterification degree was 65%. Canola oil was purchased from Qinglong Co. Ltd. (Ganzhou, China), and polystyrene latex aqueous suspension (PLAS, 10% *w*/*w* solids) was purchased from Sigma Chemical Co. (St. Louis, MO, USA). All other chemicals used in this study were of analytical grade.

### 2.2. Preparation of the O/W Emulsion

Briefly, WPC solution (20 mg/mL) was mixed with canola oil at a mass ratio of 8:2, after which the mixture was homogenized using a high-speed blender (IKA T18 basic, IKA-Werke GmbH & Co., Staufen, Germany) for 2 min at a speed of 15,000 rpm. Subsequently, the mixture was subjected to further homogenization at 30 MPa for two cycles using a high-pressure homogenizer (AMH-3, ATS Engineering Co., Ltd., Suzhou, China). Lastly, the as-prepared O/W emulsion was stored at 4 °C before use.

### 2.3. Phase Separation Products Preparation

Briefly, HWPC and HMP solutions (3%, *w*/*w*, dry weight) were prepared according to the methods reported in our previous study [3]. Subsequently, the above two solutions were mixed at a mass ratio of 3:1, and then pH was adjusted to 7.0. Thereafter, the above mixture was continuously stirred for 30 min at 1000 rpm, after which the mixtures were centrifuged at 9640× *g* rpm for 2 h at 4 °C. The upper and lower phases of the mixtures were collected, respectively.

### 2.4. Fabrication of the Filled Hydrogel

**Figure 1:**
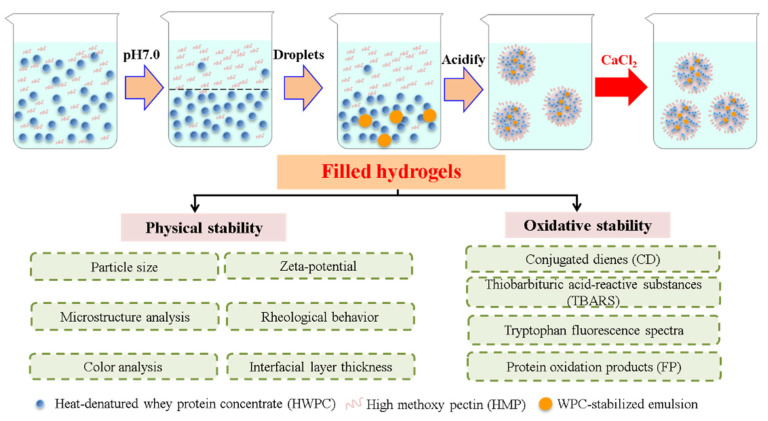
Schematic diagram of prepared filled hydrogels with different calcium chloride (CaCl_2_) levels.

Filled hydrogels were prepared based on the method by Matalanis and McClements [1] with some modifications, which are shown in Figure 1. Briefly, the as-prepared O/W emulsion, the lower phase, and upper phase were mixed at a mass ratio of 1:1:18, after which the mixture was stirred at a speed of 10,000 rpm for 45 min, while maintaining a pH of 5.0. CaCl_2_ solution (50 mM, pH 5.0) was added to as-prepared sample to achieve final Ca^2+^ levels of 0, 2, 4, 6, and 8 mM in the final systems. Lastly, sodium azide (0.02%, *w*/*w*) was added to the filled hydrogels to inhibit microbial growth.

### 2.5. Particle Size and Zeta-Potential

The particle size and surface charge of freshly prepared and 10-days-stored filled hydrogels were analyzed by using a Nano ZS dynamic light scattering instrument (Malvern instruments Ltd., Worcestershire, UK) based on the method proposed by Hinderink, Schröder, Sagis, and Berton-Carabin [17].

### 2.6. Rheological Behavior

The rheological behaviors (steady shear and dynamic viscoelastic tests) of freshly prepared filled hydrogel samples were characterized bt using a rheometer (DHR-1, TA Instruments Inc., New Castle, DE, USA). The rheometer was equipped with parallel plate geometry with a diameter and gap of 40 and 1 mm, respectively. In order to prevent water evaporation during test processing, paraffin oil was spread on the surface of the perimeter of the sample.

#### 2.6.1. Flow Behavior

The steady shear viscosity of the freshly prepared filled hydrogels was determined by increasing the shear rate from 0.1 to 100 s^−1^ at 25 °C. The flow behavior of the samples was described by fitting the Cross rheological model to the apparent viscosity (η, Pa.s)-shear rate (γ, s^−1^), which obtained data as follows:

(1) $$\frac{\eta -\eta_{\infty }}{\eta_{0}-\eta_{\infty }}=\frac{1}{1+{\left(\lambda \gamma \right)}^{m}}$$

where $\eta_{0}$ and $\eta_{\infty }$ represent the zero-shear viscosity at low shear rate and infinite-shear viscosity at high shear rate (Pa.s), respectively. λ represents the Cross model constant (s). m represents the dimensionless exponent.

#### 2.6.2. Dynamic Viscoelastic Measurements

Before measurements, linear viscoelastic sweeps were performed from 0.01 to 100% strain at a frequency of 1 Hz, and the final identified linear viscoelastic regime was 1%. A dynamic viscoelastic test was conducted in the frequency range from 1 to 100 Hz, and the viscoelastic properties (storage moduli (*G*′) and loss moduli (*G*″) and phase angle (tan *δ*, *G*″/*G*′)) of the filled hydrogels were recorded as a function of the frequency. In addition, the *G*′ or *G*″ data of the filled hydrogels were fitted to the power law model to describe the frequency dependency of samples, as indicated in Equations (2) and (3):

*log G′* = *log a′* + *b′·log f* (2)

*log G″* = *log a″* + *b″·log f* (3)

where *f* is the frequency (Hz), *a*′ and *a*″ represent the corresponding fitting parameters (Pa/s^n^), and *b*′ and *b*″ represent dimensionless frequency indices.

### 2.7. Interfacial Layer Thickness

The interfacial layer thickness of freshly prepared filled hydrogels was calculated based on the study of Wong, Day, and Augustin [18]. Briefly, 0.5 g of PLAS (0.1% *w*/*v*) and 2.0 g of WPC (2.0%, *w*/*w*) were mixed, after which 2.5 g of the lower phase and 45 g of the upper phases were added to the mixture. The systems were stirred at 10,000 rpm for 45 min, while maintaining the pH at 5.0. Subsequently, the CaCl_2_ solution (50 mM, pH 5.0) was added to the as-prepared mixture until final Ca^2+^ levels of 0, 2, 4, 6, and 8 mM in final systems were achieved. Lastly, the average particle diameter of the mixture system was determined at 25 °C.

### 2.8. Colour Measurement

The difference in the color (L* value (lightness), a* value (redness/greenness), and b* value (yellowness/blueness)) of the filled hydrogels was captured by using a ZE-6000 colorimeter (Nippon Denshoku, Kogyo Co., Tokyo, Japan). In addition, the whiteness value was calculated as follows:

(4) $$\mathrm{Whiteness}=100-\sqrt{{\left(100-L^{*}\right)}^{2\;}+a^{*}{}^{2}+b^{*}{}^{2}}$$

### 2.9. Morphology Analysis

The morphology of the freshly prepared filled hydrogels was visualized by using cryo-scanning electron microscopy (Cryo-SEM; S-3400N, Hitachi, Japan) according to the description of our previous study [19]. Images of the microstructures of the samples were captured at 30,000× magnification with a digital camera at an acceleration voltage of 5.0 kV.

### 2.10. Lipid Oxidation

The conjugated diene (CD) is the primary oxidation product of lipids in the filled hydrogel samples, which was analyzed according to the method of Corongiu and Banni [20]. Briefly, 1.5 mL of isooctane-isopropanol (3:1, *v*/*v*) was added into 0.1 mL of filled hydrogels, after which the mixture was vortexed at 3 × 10 s. After mixing, the mixture was centrifuged at a speed of 530 rpm for 2 min, after which the obtained supernatant was mixed with 4.8 mL of isooctane. Lastly, the absorbance of mixtures was determined at 234 nm.

The thiobarbituric acid-reactive substances (TBARSs) value, which is a marker for secondary lipid oxidation products, was evaluated using the method of a previous study [21]. Briefly, 2 mL of Thiobarbituric acid (TBA) reagent (15% trichloroacetic acid and 0.375% thiobarbituric acid in 0.25 M HCl) and 1 mL of filled hydrogels were mixed and then reacted, after which the mixture was heated by placing in a boiling water bath for 15 min. After cooling to room temperature, the mixture was centrifugated for 15 min at 3470 rpm, and the absorbance of the pinkish upper phase was measured at 532 nm. The TBARS values in the filled hydrogels were calculated based on the calibration curve equation prepared using 1,1,3,3-tetraethoxypropane as the standard.

### 2.11. Protein Oxidation Measurements

The protein oxidation behavior of the filled hydrogels was determined using fluorescence measurements based on the procedure of Cao et al. [19] using a 970 CRT spectrofluorimeter (970 CRT, Shanghai Precision Instrument Co., Ltd., Shanghai, China). For the natural tryptophan fluorescence, the filled hydrogel was diluted 50-fold using 10 mM phosphate buffer (pH 7.0), after which the optical density of the samples was recorded in the spectral range from 300 to 400 nm at a fixed excitation wavelength of 283 nm. For the tryptophan fluorescence of protein oxidation products (FP), the fluorescence range was set in the range from 400 to 500 nm at an excitation of 350 nm.

### 2.12. Statistical Analyses

Three batches of filled hydrogels (replicates) treated with different concentrations of CaCl_2_ were prepared, and triplicate measurements and analyses of related characteristics were performed for each replicate. Data were analyzed using the General Linear Models procedure of the Statistix 8.1 software package (Analytical Software version 8.1, St. Paul, MN, USA), and the results were expressed as mean ± standard deviations (SD). The significance of the main effects was evaluated using a one-way analysis of variance (ANOVA) with Tukey’s multiple comparison procedure (*p* < 0.05). Correlation analysis between the physical and oxidative properties of the as-prepared filled hydrogels was performed using the R-3.5.3 software (version 3.5.3, Tsinghua University, Beijing, China).

## 3. Results and Discussion

### 3.1. Particle Size

The average diameter of emulsion droplets is one of the important indicators for evaluating the emulsion stability of filled hydrogels, and the average diameters of the prepared filled hydrogels are shown in Table 1. The size of the droplets in the freshly prepared filled hydrogels ranged from 24.97 to 28.40 µm. Compared with the filled hydrogel without CaCl_2_, there was a significant increase in the average diameter of the droplets of the filled hydrogels containing CaCl_2_ (*p* < 0.05). This was attributed to the fact that the strong binding affinity between Ca^2+^ and the whey protein/pectin absorbed on the interface increased the degree of aggregation of the droplets, which resulted in the formation of relatively larger droplets [1,15,16]. Nevertheless, no significant difference in the average diameter of the droplets in the freshly prepared filled hydrogels with different CaCl_2_ levels was observed (2–8 mM, *p* > 0.05), suggesting that CaCl_2_ levels had no significant effects on the size of the oil droplets. Similar results are observed in the results reported by Du et al. [15], in which the presence of Ca^2+^ had minor significant effect on the particle size of high-calorie whey protein emulsions.

After 10 days storage reaction, the average diameters of the oil droplets in the samples were larger than those of the freshly prepared filled hydrogels. This increase may be attributed to the coalescence caused by Brownian motion collision [22]. Moreover, compared with control group (without CaCl_2_), the addition of lower level of CaCl_2_ (2 or 4 mM) obviously increased particle size and decreased the increasing rate of the average diameter versus time (*p* < 0.05), indicating that appropriate CaCl_2_ levels improve physical stability during storage. However, the average diameters of oil droplets in the stored filled hydrogel samples gradually increased with continually increasing CaCl_2_ levels from 6 to 8 mM, and the increasing rate of the average diameter versus time also gradually increased. This was attributed to the extensive aggregation of all oil droplets at higher Ca^2+^ levels. A similar phenomenon was observed in the study of Keowmaneechai and McClements [23], who reported that a further increase in CaCl_2_ concentration above the critical concentration resulted in extensive droplets aggregation, which decreased the relative stability of the emulsion.

### 3.2. Zeta-Potential

The zeta-potential was used to evaluate the local charge distribution and net surface charge of lipid droplets in the filled hydrogels to analyze the stability of the filled hydrogels [24]. The freshly prepared filled hydrogels exhibited a negative surface charge in the range from −34.30 to −32.00 mV (Table 2). This could be attributed to the negative charge of whey protein at a pH of 5.0 (which is above the isoelectric point of protein) and the negative charge of pectin at all pH ranges. The addition of CaCl_2_ into the freshly prepared filled hydrogels significantly increased absolute zeta-potential values of the samples (*p* < 0.05). The increased zeta-potential of the samples containing CaCl_2_ corresponded to an enhancement in the physical stability of the filled hydrogels [25]. However, the absolute zeta-potential values of the filled hydrogel samples containing CaCl_2_ levels of 6 and 8 mM were lower than those of the other filled hydrogel samples, indicating that higher CaCl_2_ levels reduced the repulsive force between the droplets and induced extensive calcium-mediated protein aggregation, which resulted in the physical instability of the filled hydrogels. The Ca^2+^ present in the filled hydrogels easily combined with the free carboxyl groups of the proteins to form ionic bridges between proteins by reducing electrostatic repulsions between proteins, which decreased the negative charge on the surface of the droplets [15,26]. In addition, ionic binding caused a reduction in surface charge. After a 10-day incubation period, there was a significant decrease in negative surface charges of the droplets of the filled hydrogels, which could be attributed to an imbalance of droplets and the water phase throughout the storage, which increased the instability of the filled hydrogels.

### 3.3. Apparent Viscosity

The steady shear behavior of the filled hydrogel samples prepared in this study is shown in Figure 1. With or without the presence of CaCl_2_, all filled hydrogel samples exhibited typical shear thinning flow behaviors. Li et al. [27] reported that shear thinning behavior is highly related to changes in molecular configurations during the shearing process owing to the formation of additional chains in the molecules. Steady shear flow curves were well fitted with the Cross model, which was due to possessing a correlation coefficient (R^2^) above 0.95 for each sample. Dimensionless exponents (m) were employed to understand the effect of shear on the filled hydrogels, and the m values of the hydrogel samples are shown in Table 3. The m values of the filled hydrogels were less than 1, which further verified shear-thinning rheological behavior of the filled hydrogel samples.

**Figure 2:**
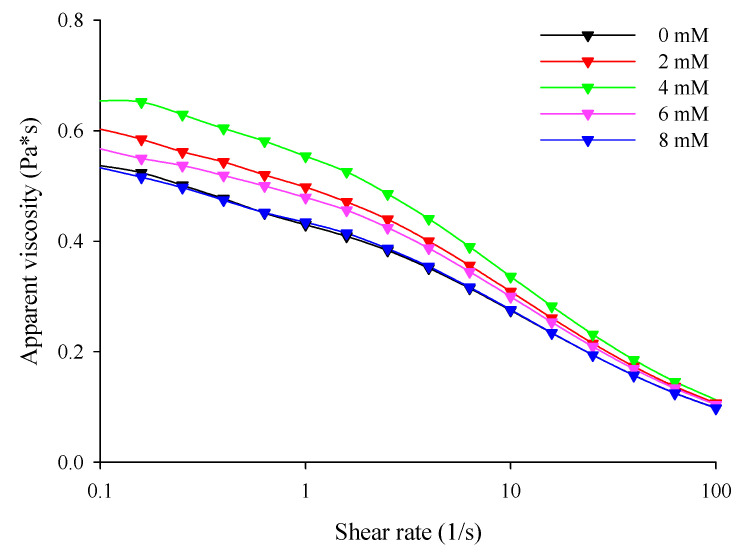
Apparent viscosity of the freshly prepared filled hydrogels containing different calcium chloride (CaCl_2_) levels.

With an increase in CaCl_2_ levels (0–4 mM), the zero-shear viscosity ($\eta_{0}$) of the filled hydrogels increased significantly (*p* < 0.05). This means that the incorporation of Ca^2+^ increased the resistance of the hydrogel to flow (Table 3). Meanwhile, the flow curve revealed that the filled hydrogels containing CaCl_2_ exhibited higher apparent viscosity than that without CaCl_2_, and the apparent viscosity of the filled hydrogels increased with increasing CaCl_2_ levels (Figure 2). This phenomenon is similar to that observed in the study of Matalanis and McClements [1], who reported that CaCl_2_ increased the apparent viscosity of filled hydrogels fabricated using HMP and sodium caseinate. When CaCl_2_ was added into the filled hydrogels, the Ca^2+^ distributed in the continuous phase promoted crosslinking between the carboxyl and ester phosphate groups present on the HWPC or HMP molecules on the surface of the droplets, thus enhancing the apparent viscosity of the filled hydrogels [15]. However, at CaCl_2_ levels above 4 mM, $\eta_{0}$ decreased, and the apparent viscosity decreased (*p* < 0.05). This phenomenon could be attributed to the fact that the excessive bridging effect induced by Ca^2+^ resulted in the excessive aggregation of proteins and the flocculation of droplets, thus having effects on the apparent viscosity of the sample [28]. Additionally, the time constant (λ) of the samples at different CaCl_2_ levels further verified the apparent viscosity results of the sample.

### 3.4. Dynamic Rheological Behavior

**Figure 3:**
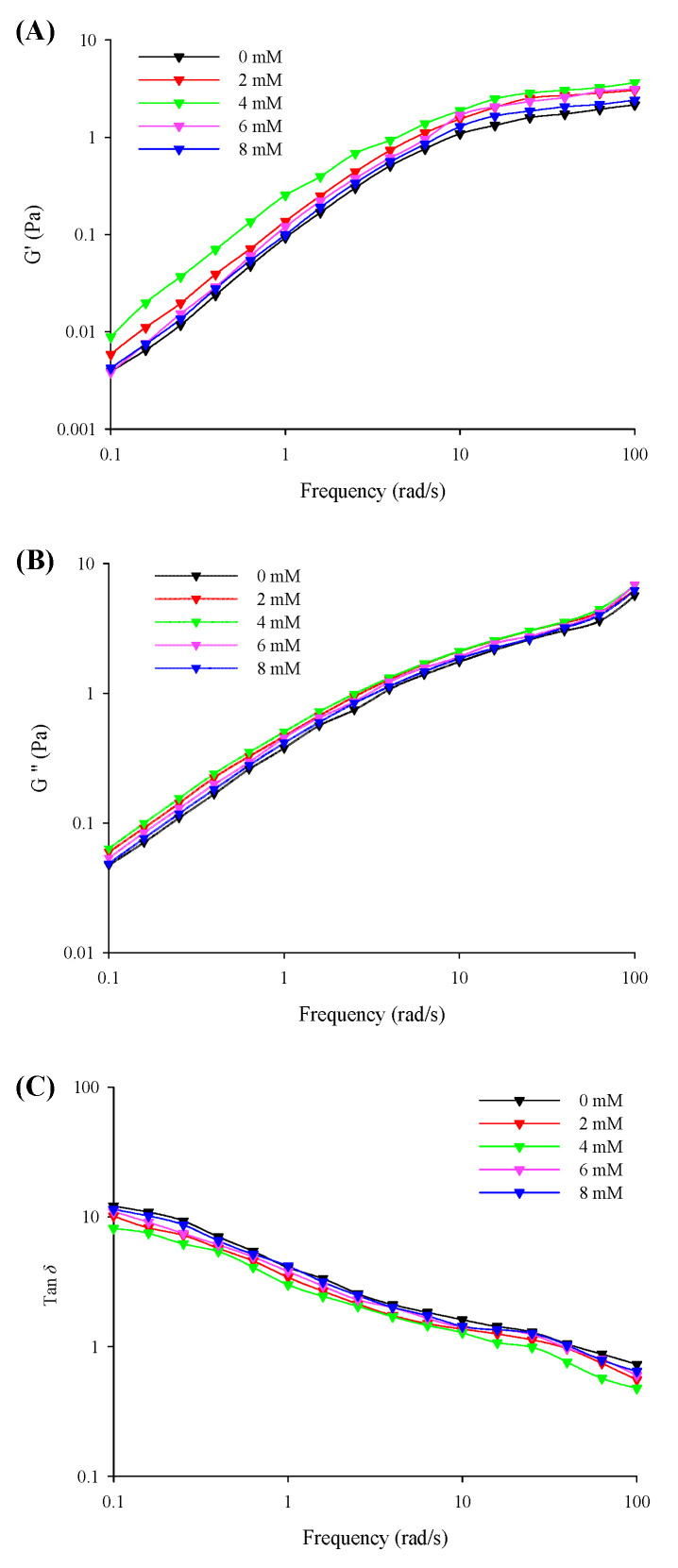
Storage moduli (*G*′, **A**), loss moduli (*G*″, **B**), and phase angle (tan *δ*, *G*″/*G*′, **C**) of the freshly prepared filled hydrogels containing different CaCl_2_ levels as a function of frequency.

The viscoelasticities (*G*′, *G*″, and tan *δ*) of the filled hydrogels with different CaCl_2_ levels under small amplitude oscillations with a change in the frequency are shown in Figure 3. The results revealed that the *G*′ was considerably lower than *G*″ (tan *δ* > 1) at the low frequency range from 0.1 to 10 Hz and was higher than *G*″ (tan *δ* < 1) with a further increase in the frequency sweep range from 10 to 100 Hz. This corresponded to the formation of solid-related and elastic gel-like properties in the filled hydrogels at low and high frequency ranges, respectively. The *G*′ and *G*″ values of the filled hydrogel samples exhibited an increasing trend with an increase in CaCl_2_ levels (0–4 mM), and the point of tan *δ* = 1 shifted to lower frequency values. Moreover, the results of the frequency dependence of the filled hydrogels revealed that the *k*′ and *k*″ values significantly increased with increasing CaCl_2_ levels (Table 4), which supported the results of the above-mentioned viscoelastic behavior. This could be attributed to the increase in crosslinking between Ca^2+^ and whey protein molecules [29]. However, a further increase in CaCl_2_ levels resulted in a gradual decrease in the *G*′ and *G*″ values of the samples and a shift in the point of tan *δ* = 1 to a higher frequency, as well as a gradual decrease in the *k*′ and *k*″ values. This could be attributed to the excessive aggregation of proteins and the flocculation of droplets. The excessive crosslinking between proteins on the surface of the droplets impaired the chemical bond within the network and resulted in the formation of heterogeneous structures, which decreased the elasticity behavior of the filled hydrogels [30]. Moreover, this could be attributed to the fact that the repulsive electrostatic force induced by the addition of Ca^2+^ reduced the strength of the interface film, thereby declining the viscoelasticity of the oil-in-water interface [31]. In addition, n′ and n″ results revealed that lower CaCl_2_ levels decreased the frequency sensitivity of filled hydrogels, whereas lower CaCl_2_ levels enhanced frequency sensitivity relative to the frequency of filled hydrogels. A reduction in the sensitivity of the hydrogels to frequency enabled the formation of weak elastic gel-like structures and improved the stability of filled hydrogels.

### 3.5. Color Analysis

The instrumental colour values, which affect the acceptance and choice by consumers, of the filled hydrogel samples in terms of the L*, a*, and b* values are presented in Table 5. The L* value of the filled hydrogel samples without CaCl_2_ could be attributed to the light scattering of the droplets. Compared to those of the hydrogel samples without CaCl_2_, the L* values of the hydrogel samples containing CaCl_2_ decreased significantly with increasing CaCl_2_ levels (*p* < 0.05). This could be attributed to the fact that the incorporation of Ca^2+^ increased crosslinking between proteins and pectin, which reduced the refractive index and scattering efficiency compared to those of the surrounding aqueous phase. This result was consistent with the results of particle size of the filled hydrogels with increasing CaCl_2_ levels. Moreover, no significant differences in a* and b* values of the filled hydrogels with different CaCl_2_ levels were observed, indicating that the incorporation of Ca^2+^ had no effect on the redness and yellowness of the filled hydrogel samples. Furthermore, with an increase in the CaCl_2_ levels, the a* and b* values of the filled hydrogel sampled first increased and then decreased, which was consistent with L* value results.

### 3.6. Interfacial Layer Thickness

**Figure 4:**
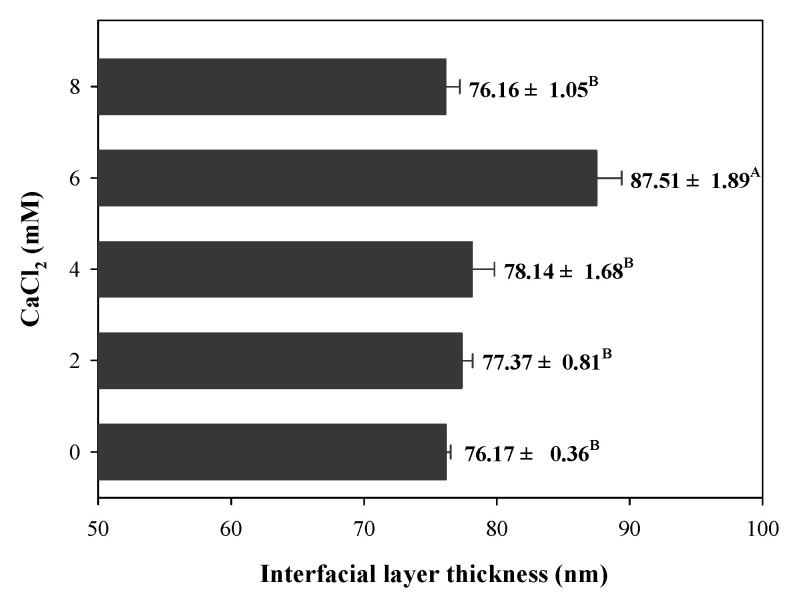
Interfacial layer thickness of freshly prepared filled hydrogels containing different CaCl_2_ levels. The different letters (A, B) represent significant differences among filled hydrogels at different CaCl_2_ levels (*p* < 0.05).

During the filled hydrogels formation process, protein or polysaccharide molecules were adsorbed on the surface of the droplets to form an interfacial film layer, which significantly affects the stability of the filled hydrogel. Therefore, in this study, we evaluated the interfacial layer thickness of filled hydrogels containing different CaCl_2_ levels. Owing to the binding between the filled hydrogels and CaCl_2_, the samples containing CaCl_2_ exhibited a significantly larger interfacial layer thickness than the filled hydrogels without CaCl_2_ (*p* < 0.05, Figure 4). The increase in interfacial layer thickness of the filled hydrogel containing CaCl_2_ could be attributed to the adsorption of proteins to the surface of the droplets. Taha et al. [32] indicated that the addition of divalent cations promoted protein aggregation and electrostatic shielding effects, which significantly enhanced the adsorption of proteins at the interface. Moreover, Guo et al. [29] reported that the interaction between protein and Ca^2+^ enhanced the dilatational properties and entanglements of adsorbed protein at the oil–water interface, which significantly affected the interfacial layer thickness. However, with a further increase in CaCl_2_ levels (8 mM), the interfacial layer thickness decreased. These results indicate that excessive Ca^2+^ levels might result in higher degree of droplet aggregations, which decreases the stabilization of adsorbed particles located at oil–water interfaces [29].

### 3.7. Surface Characterization of Droplets

**Figure 5:**
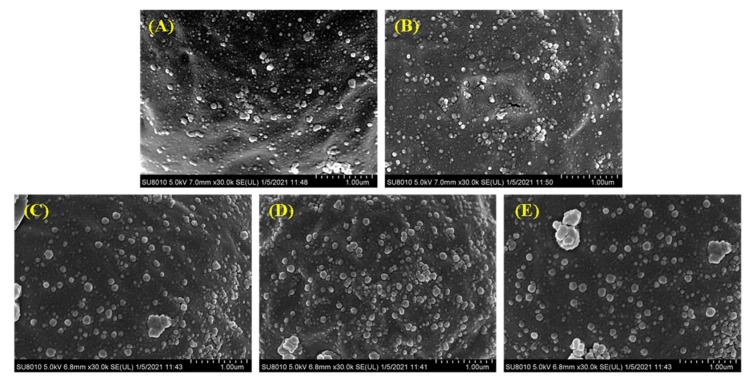
Cryo-scanning electron microscopy (SEM) images of the freshly prepared filled hydrogels containing different CaCl_2_ levels. (**A**–**E**) correspond to images of filled hydrogels sample containing CaCl_2_ levels of 0, 2, 4, 6, and 8 mM.

The surface of the droplets of the filled hydrogels under different CaCl_2_ levels was characterized by using SEM. SEM images revealed that, in the absence of CaCl_2_, the droplets of the filled hydrogels were coated by the interfacial film incorporated by whey proteins and HMP. Moreover, the molecules on the interface of droplets were uniformly dispersed and exhibited a relatively small particle shape (Figure 5A). The stabilization of the filled hydrogels fabricated using the whey protein–HMP complex could be attributed to the intermixed layer of whey protein and HMP or the combination of an inter-polyelectrolyte network formed by linking pectin chains with clustered proteins [33]. Moreover, the incorporation of CaCl_2_ increased the adsorption capacity of proteins/polysaccharides on the water–oil interface (Figure 5B–D). Meanwhile, with an increase in CaCl_2_ levels, the size and thickness of the aggregated particles at the droplets interface increased. The incorporation of Ca^2+^ resulted in the binding of Ca^2+^ to the anionic groups on the surfaces of the droplets, thereby enabling a reduction in the distance between them [34]. Moreover, the presence of Ca^2+^ enhanced the adsorption capacity of protein at the interface [32], which hindered the deformation of the interface layer with an enhancement of filled hydrogels. This result was consistent with interfacial layer thickness results. However, at higher CaCl_2_ levels (such as 6 and 8 mM), there was a significant increase in the number of aggregation particles at the interface, and the particles were gradually transformed into irregularly shaped particles with relatively large cloud-like aggregates. In addition, there was a decrease in the number of interface particles (Figure 5E), indicating a reduction in the stability of filled hydrogels. Excessive charge neutralization and ion bridging effects caused by Ca^2+^ promoted the formation of larger aggregates, resulting in a shift in the location of the protein molecules from the surface of the droplets into the continuous phase [35].

### 3.8. Lipid Oxidation

**Figure 6:**
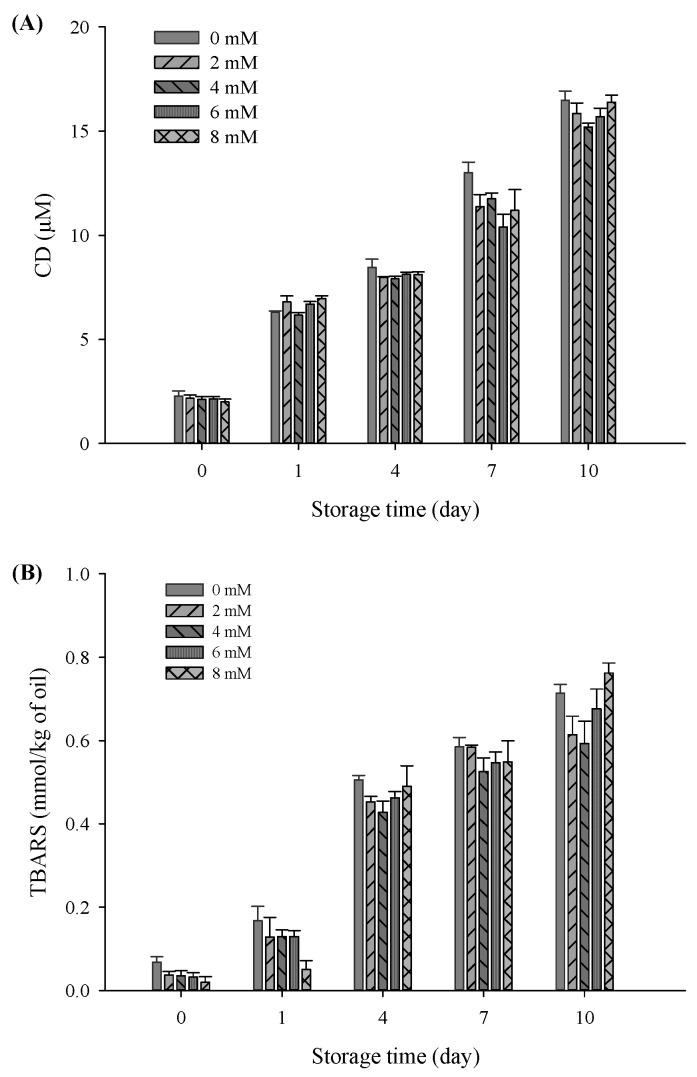
Conjugated diene (**A**) and thiobarbituric acid-reactive substances; (**B**) values of the filled hydrogel with different CaCl_2_ levels at storage time at 37 °C.

In this study, CD and TBARS were used as indicators to evaluate the effects of CaCl_2_ addition on lipid oxidation properties of the filled hydrogel samples during a storage period of 10 days. There was a significant increase in CD and TBARS values of all samples with an increase in the storage time (*p* < 0.05), verifying the occurrence of lipid oxidation during storage (Figure 6). Moreover, there was a sudden decrease in the CD of the freshly prepared filled hydrogels containing CaCl_2_ (*p* < 0.05), which was in accordance with TBARS value results. After incubation for 10 days, CD and TBARS values obviously decreased and then increased with increasing CaCl_2_ levels (*p* < 0.05), and the lowest CD and TBARS values were observed at CaCl_2_ levels of 4 mM. This suggested that appropriate Ca^2+^ levels effectively inhibited lipid oxidation. A previous study demonstrated that protein in emulsions protects oil against oxidation, which was due to the ability of scavenging free radicals, chelating metal ions, and increase in thickness of the viscoelastic interfacial layer [36]. When Ca^2+^ was added to the filled hydrogel samples, there was an increase in the amount of protein adsorbed on the surface of the droplets and the interfacial layer thickness of the droplets, thereby improving the oxidative stability of the emulsion via the prevention of the migration of transition metal ions to core lipids [37]. These results are in accordance with the increase in the interfacial layer thickness results with increasing CaCl_2_ levels. Moreover, due to electrostatic bridges between proteins and Ca^2+^, there was an increase in the number of protein molecules in the dispersion phase adsorbed on the surface of droplets, which enhanced the antioxidant capacity of proteins [32]. In addition, the higher continuous phase viscosity of samples observed in the steady shear rate measurement was one of the major factors involved in the decrease in the diffusion of oxidants relative to the oil droplet’s surface area, thus improving the lipid oxidative stability of the filled hydrogels [38]. However, electrostatic shielding induced by higher Ca^2+^ levels (exceed 4 mM) resulted in multiple aggregations or flocculation of droplet, which accelerated the rate of lipid oxidation and resulted in higher CD and TBARS values [39]. The aforementioned results indicated that an appropriate Ca^2+^ level can remarkably prevent the formation of oxidative lipid products, in which low CD and TBARS levels are chief lipids oxidation markers.

### 3.9. Protein Oxidation

**Figure 7:**
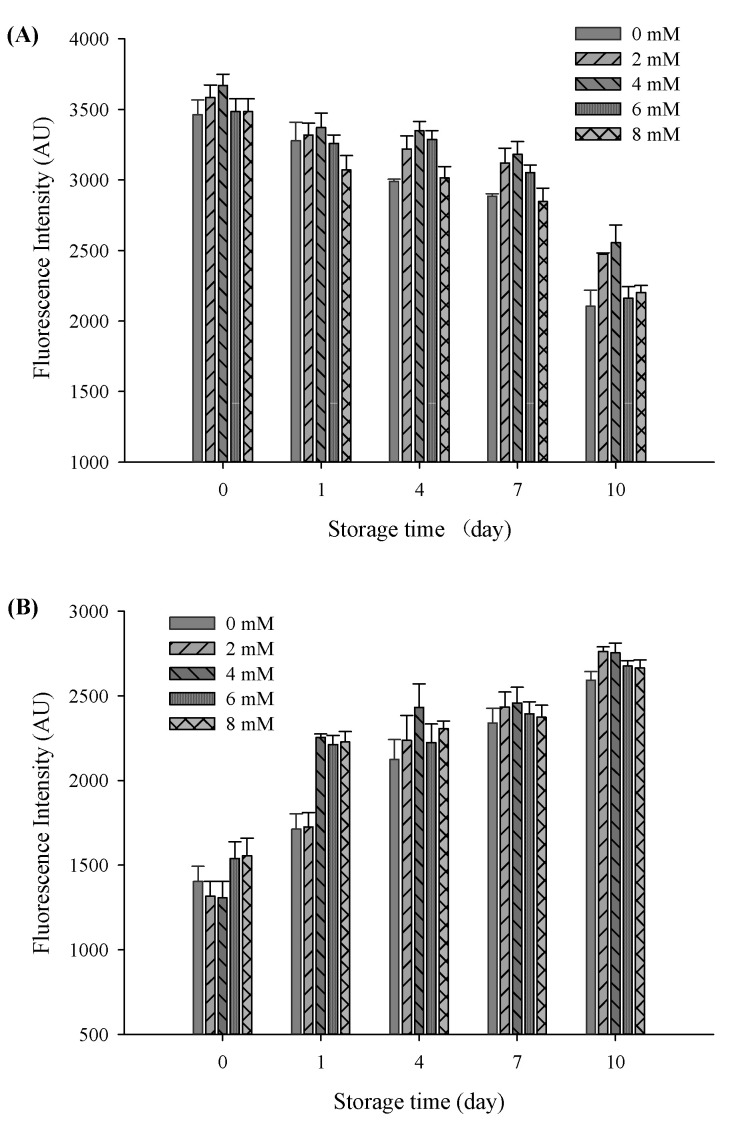
Tryptophan fluorescence spectra (**A**) and FP fluorescence spectra (**B**) of the filled hydrogel system containing different CaCl_2_ levels at different storage times at 37 °C.

The oxidative stability of filled hydrogels containing CaCl_2_ for the influence of protein oxidation was compared to those of the samples without CaCl_2_. To this end, the protein oxidative stability of filled hydrogels at different CaCl_2_ levels was measured. A decrease in the intrinsic fluorescence of tryptophan is considered as a vital parameter for illustrating protein oxidative stability. At a storage time of 0 days, the intensity of tryptophan fluorescence gradually increased with increasing CaCl_2_ levels, and the highest intensity value was observed at a CaCl_2_ level of 4 mM (*p* < 0.05, Figure 7A). In addition, the incorporation of CaCl_2_ induced the interaction of Ca^2+^ with free anionic carboxyl groups of proteins, thus increasing the intrinsic fluorescence of tryptophan. However, tryptophan fluorescence decreased with a further increase in CaCl_2_ levels to 8 mM. The reduction in tryptophan fluorescence was mainly due to excessive protein aggregation and flocculation of droplets, which resulted in a decrease in the exposure of tryptophan. After subjecting the filled hydrogels to storage for 10 days, the intensity of the tryptophan fluorescence of the filled hydrogels decreased continuously (*p* < 0.05), which revealed that the protein oxidation occurred throughout the storage period [40]. Fluorescence quenching during storage might be due to protein modification and crosslinking caused by oxidation, which reduced the exposure possibility of tryptophan [41]. After storage for 10 days, with the increase in CaCl_2_ levels, the intensity of tryptophan fluorescence increased and then decreased (*p* < 0.05), and the highest fluorescence value was observed at a CaCl_2_ level of 4 mM. These results indicated that an appropriate Ca^2+^ level can remarkably prevent the oxidation of proteins, and excessive Ca^2+^ levels can accelerate the rate of proteins oxidation throughout the entire storage time.

As the secondary protein oxidation products, the yield of FP is used to evaluate the extent of protein oxidation. The fluorescence emitted by the FP of all filled hydrogels increased gradually throughout the storage period (*p* < 0.05, Figure 7B). Similar to the loss of tryptophan fluorescence, the increase in the fluorescence of the hydrogel samples over time could be attributed to the interaction between oxidized proteins and secondary lipid oxidation products [42]. Moreover, with an increase in CaCl_2_ levels, the fluorescence intensity of filled hydrogel samples significantly increased and then decreased at certain storage times (*p* < 0.05). In addition, the tertiary protein structures of the sampled altered the antioxidative capacity of protein [43]. The addition of CaCl_2_ effectively shielded protein against oxidation, as indicated by the increase in fluorescence intensity of the filled hydrogels. However, with a further increase in CaCl_2_ level to 6 and 8 mM, fluorescence intensity decreased, which could be mainly ascribed to the aggregation of FP accelerating protein oxidation.

### 3.10. Correlation between Physical and Oxidative Stability

**Figure 8:**
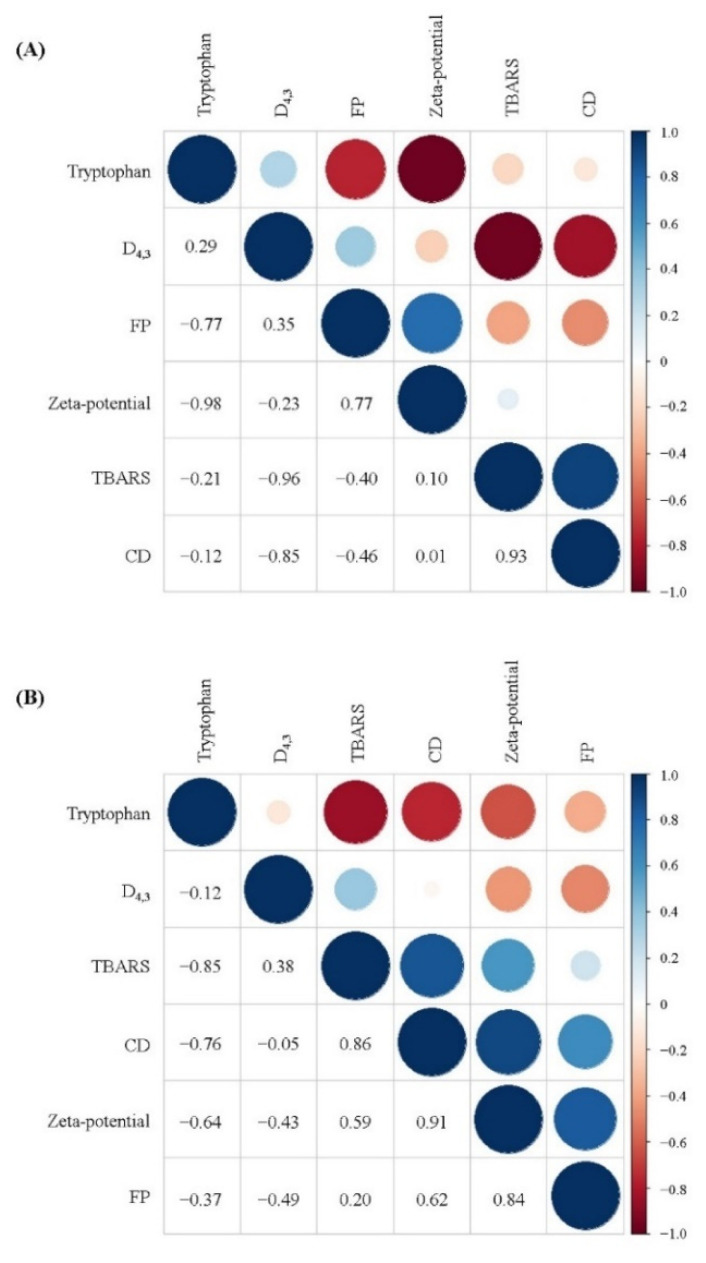
Correlation analysis between the physical and oxidative stability of filled hydrogels containing different CaCl_2_ levels for a storage time of 0 days (**A**) and 10 days (**B**).

As shown in Figure 8, correlation analysis showed that the particles size (*D*_4_._3_) of the freshly prepared filled hydrogels exhibited a high negative correlation with CD and TBARS values (R = −0.85 and R = 0.96), whereas the zeta-potential exhibited a slight positive correlation with CD and TBARS values (R = 0.01 and R = 0.10). These results are consistent with those of a previous study that reported that the particle size of droplets exhibited a negatively correlation with the TBARS value of prepared filled hydrogels. Moreover, the CD and TBARS values of the freshly prepared filled hydrogels exhibited a negative correlation with tryptophan fluorescence intensity, indicating that the oxidation of lipids of the filled hydrogels had a significant effect on the oxidation of proteins. This result was similar to that observed by Estévez et al. [42], who reported that there was a strong negative linear correlation between tryptophan emulsion fluorescence and primary oxidative products. Lipid oxidation products promoted protein oxidation during the oxidation process [44], which could be ascribed to the fact that lipid-derived oxidative products reacted with amino acids/peptides/proteins, thereby resulting in various protein oxidative products. Additionally, unabsorbed proteins in the aqueous phase or adsorbed protein at the interfacial layer affected the lipids oxidation [45,46]. In addition, after incubation for 10 days, the positive correlation between the lipid oxidation values (CD and TBARS values) and protein oxidation values (tryptophan fluorescence intensity and FP) increased. This indicated that the intensity of the extent of protein and lipids oxidation increased, and the interaction between protein and lipid oxidation became more significant with increasing storage time.

## 4. Conclusions

In summary, our present study revealed that CaCl_2_ levels had significant effects on the stabilities of the filled hydrogels. CaCl_2_ addition enhanced the physical stability of the filled hydrogels sample, which could be due to the strong binding affinity between Ca^2+^ and whey protein/pectin mixtures, which were absorbed at the interface, thereby increasing the degree of aggregation and electrostatic shielding effects in the samples. Moreover, appropriate Ca^2+^ levels (4 mM) significantly enhanced the oxidative stability of the filled hydrogels, which could be potentially applied as an innovative delivery system to encapsulate bioactive compounds, as well as a reduced calorie low-fat food product. This phenomenon was attributed to the increase in protein molecules adsorbed on the surface of the droplets and the increase in the interfacial layer thickness of the droplets by the addition of Ca^2+^, which hindered the migration of the transition metal ions to the core lipids and the antioxidant capacity of proteins. However, excessive Ca^2+^ levels (6 or 8 mM) resulted in a higher degree of droplet aggregations and a subsequent decrease in the stabilization of adsorbed particles located in droplets’ interfaces, which negatively affected the oxidative stability of the filled hydrogels. Further research will focus on investigating the effect of different Ca^2+^ levels on the in vitro digestibility of filled hydrogels in the gastrointestinal digestive system.

## Figures and Tables

**Table 1:** Droplet size of filled hydrogels containing different CaCl_2_ levels for fixed storage times (0 and 10 days).

CaCl_2_ (mM)	The Volume Averaged Diameter (D_4,3_, μm)		Increased Rate (%)	The Surface-Averaged Diameter (D_3,2_*,* μm)		Increased Rate (%)
	Day 0	Day 10		Day 0	Day 10	
0	24.97 ± 0.93 ^B^	31.64 ± 0.95 ^C^	26.74 ± 0.91 ^A^	5.40 ± 0.05 ^C^	10.13 ± 0.07 ^D^	87.60 ± 0.44 ^A^
2	27.23 ± 0.54 ^AB^	33.10 ± 0.67 ^BC^	21.56 ± 0.05 ^B^	5.76 ± 0.03 ^B^	10.39 ± 0.10 ^C^	80.38 ± 0.80 ^C^
4	27.96 ± 0.80 ^A^	33.51 ± 0.96 ^BC^	19.85 ± 0.01 ^B^	5.81 ± 0.05 ^B^	10.66 ± 0.06 ^B^	83.48 ± 0.55 ^B^
6	28.31 ± 1.13 ^A^	35.05 ± 0.44 ^AB^	21.35 ± 2.19 ^B^	5.98 ± 0.04 ^A^	10.91 ± 0.10 ^A^	82.44 ± 0.45 ^B^
8	28.40 ± 0.99 ^A^	36.06 ± 1.34 ^A^	26.97 ± 0.29 ^A^	6.03 ± 0.07 ^A^	11.02 ± 0.12 ^A^	82.75 ± 0.13 ^B^

Values are expressed as mean ± SD from triplicate measurements. A–D in each column represent statistically significant differences (*p* < 0.05).

**Table 2:** Zeta-potential of the filled hydrogels containing different CaCl_2_ levels for fixed storage times (0 and 10 days).

CaCl_2_ (mM)	Zeta-Potential (mV)	
	Day 0	Day 10
0	−32.13 ± 0.51 ^B^	−26.65 ± 0.14 ^C^
2	−33.10 ± 0.27 ^AB^	−27.83 ± 0.60 ^BC^
4	−34.30 ± 0.53 ^A^	−29.38 ± 0.49 ^A^
6	−32.40 ± 0.69 ^B^	−28.58 ± 0.09 ^AB^
8	−32.00 ± 0.70 ^B^	−27.86 ± 0.77 ^BC^

Values are expressed as mean ± SD from triplicate measurements. A–C in each column represents statistically significant differences (*p* < 0.05).

**Table 3:** Steady shear parameters of the freshly prepared filled hydrogels containing different CaCl_2_ levels calculated using the Cross model.

CaCl_2_ (mM)	Zero-Shear Viscosity $\left(\eta_{0}\right)$	The Time Constant (λ)	The Dimensionless Exponent (m)	The Correlation Coefficient (R^2^)
0	0.5364 ± 0.0015 ^D^	0.2365 ± 0.0006 ^A^	0.4742 ± 0.0011 ^D^	0.9876
2	0.6035 ± 0.0006 ^B^	0.1258 ± 0.0009 ^C^	0.6037 ± 0.0009 ^B^	0.9968
4	0.6543 ± 0.0022 ^A^	0.0817 ± 0.0007 ^D^	0.8254 ± 0.0010 ^A^	0.9774
6	0.5683 ± 0.0013 ^C^	0.1534 ± 0.0006 ^B^	0.5265 ± 0.0014 ^C^	0.9878
8	0.5323 ± 0.0012 ^E^	0.2385 ± 0.0001 ^A^	0.4677 ± 0.0010 ^E^	0.9829

Values are expressed as mean ± SD from triplicate measurements. A–E in each column represent statistically significant differences (*p* < 0.05).

**Table 4:** Frequency sweep of the storage moduli (*G*′) and loss moduli (*G*″) of the freshly prepared filled hydrogels containing different CaCl_2_ levels using the power law model.

CaCl_2_ (mM)	*log G*′ *= log a*′ *+ b*′·*log f*			*log G*″ *= log a*″ *+ b*″·*log f*		
	The Model Parameters (*a*′)	The Dimensionless Frequency Indices (*b*′)	R^2^	The Model Parameters (*a*″)	The Dimensionless Frequency Indices (*b*″)	R^2^
0	0.0784 ± 0.0039 ^E^	1.2806 ± 0.0012 ^B^	0.9934	0.3170 ± 0.0211 ^C^	0.6695 ± 0.0014 ^A^	0.9764
2	0.1191 ± 0.0021 ^B^	1.2493± 0.0023 ^C^	0.9940	0.3952 ± 0.0247 ^AB^	0.6490 ± 0.0021 ^C^	0.9745
4	0.1877 ± 0.0034 ^A^	1.1666 ± 0.0025 ^D^	0.9830	0.4186 ± 0.0123 ^A^	0.6412 ± 0.0019 ^D^	0.9756
6	0.0976 ± 0.0037 ^C^	1.3280 ± 0.0019 ^A^	0.9944	0.3655± 0.0302 ^ABC^	0.6631 ± 0.0023 ^B^	0.9763
8	0.0882± 0.0027 ^D^	1.2836 ± 0.0011 ^B^	0.9948	0.3384 ± 0.0124 ^BC^	0.6698 ± 0.0027 ^A^	0.9759

Values are expressed as mean ± SD from triplicate measurements. A–E in each column represent statistically significant differences (*p* < 0.05).

**Table 5:** Color of the freshly prepared filled hydrogels containing different CaCl_2_ levels.

CaCl_2_ (mM)	L* Value	a* Value	b* Value	Whiteness
0	59.51 ± 0.54 ^A^	−2.10 ± 0.01 ^A^	−2.45 ± 0.21 ^A^	59.67 ± 0.04 ^A^
2	59.40 ± 0.28 ^A^	−2.17 ± 0.02 ^A^	−2.51 ± 0.16 ^A^	59.23 ± 0.11 ^AB^
4	57.82 ± 0.61 ^B^	−2.21 ± 0.07 ^A^	−2.62 ± 0.10 ^A^	58.34 ± 0.11 ^C^
6	56.73 ± 0.56 ^BC^	−2.28 ± 0.09 ^A^	−2.68 ± 0.02 ^B^	57.67 ± 0.07 ^C^
8	55.89 ± 0.31 ^C^	−2.31 ± 0.05 ^A^	−2.69 ± 0.06 ^A^	56.09 ± 0.25 ^D^

Values are expressed as mean ± SD from triplicate measurements. A–D in each column represent statistically significant differences (*p* < 0.05).

## Data Availability

The data presented in this study are available in the article.
